# Stable in Biocompatible Buffers Silver Nanoisland Films for SERS

**DOI:** 10.3390/bios11110448

**Published:** 2021-11-12

**Authors:** Alexey Skvortsov, Ekaterina Babich, Alexey Redkov, Andrey Lipovskii, Valentina Zhurikhina

**Affiliations:** 1 Institute of Biomedical Systems and Biotechnology, Peter the Great St. Petersburg Polytechnic University, Polytechnicheskaya 29, 195251 St. Petersburg, Russia; colbug@mail.ru; 2 Institute of Physics and Mechanics, Peter the Great St. Petersburg Polytechnic University, Polytechnicheskaya 29, 195251 St. Petersburg, Russia; lipovskii@mail.ru (A.L.); jourikhina@mail.ru (V.Z.); 3 Institute of Problems of Mechanical Engineering, Bolshoy pr. V. O. 61, 199178 St. Petersburg, Russia; red-alex@mail.ru

**Keywords:** ion-exchange, silver nanoparticles, stability, luminescence, Raman, SERS, PBS

## Abstract

We investigated the stability of silver nanoisland films, which were formed on glass surface by the method of out-diffusion, in biocompatible buffers and the applicability of the films in surface enhanced Raman scattering (SERS). We have shown that silver nanoisland films are stable in one of the most widespread in biological studies buffer—phosphate buffer saline (PBS), and in 1:100 water-diluted PBS, in the PBS-based buffer, in which NaCl is replaced by the same amount of NaClO_4_, and in acidic phosphate buffer. At the same time, the replacement of NaCl in PBS by N(CH_3_)_4_Cl leads to the degradation of the nanoislands. It was shown that after exposure to PBS the nanoisland films provided a good SERS signal from a monolayer of 1,2-di(4-pyridyl)ethylene (BPE), which makes silver nanoisland films promising for biosensor applications. Additionally, in our experiments, we registered for the first time that silver nanoparticles formed in the bulk of the samples dissolved after exposing to PBS, while nanoislands on the glass surface stayed unchanged. We associate this phenomenon with the interaction of ions contained in PBS solution with silver, which results in the shift of corresponding chemical equilibrium.

## 1. Introduction

Surface plasmon resonance (SPR) in noble metal nanoparticles (NPs) makes them a very powerful tool for biosensing applications. Since the environment in the near field of a plasmon NPs can sufficiently change the SPR position, the analyte concentration and interactions of biomolecules can be detected in real time with a high resolution. SPR biosensors were effectively used for real-time biotin-streptavidin affinity measurements [1], detection of single non-absorbing molecules [2], measurements of protein–protein and protein–DNA interactions [3], and many others [4]. Surface enhanced Raman scattering (SERS), based on the enhancement of incident and scattered electric fields in the vicinity of NPs at the SPR excitation, helps to increase the sensitivity for detecting biological analytes at very low concentrations [5,6,7]. However, in SERS bio-experiments one needs to assure the reliability of measurements, since the substrates with noble metal NPs made in different ways can behave differently in biological solutions and react with biological objects. One of the most promising SERS substrates are nanoisland films of noble metals, which can be made by depositing or growth of metal nanoparticles on the surface [8,9], annealing of a thin metal film [10,11] or out-diffusion of metal from glass substrates [12,13]. An important feature of such substrates, which makes them attractive for use, is their uniformity, which provides uniform enhancement of Raman signal over the substrate, and fabrication reproducibility [9,14]. However, despite the widespread use of SERS substrates with nanoisland films, their stability in biological buffers used in most experiments has been studied insufficiently. Up to the authors’ knowledge, only one work is devoted to such a study: recently, L. Zhou and R. Ionescu reported on destabilization of Au NPs, obtained via annealing of a thin gold film, in phosphate buffer saline (PBS) [15]. We have studied the influence of several buffers, first of all, the most commonly used one in biological research-PBS, on silver nanoisland films, obtained by a different technique-out-diffusion of silver atoms from a silver ions-enriched glass substrate under annealing in hydrogen atmosphere [16]. The research was aimed at testing stability of the nanoislands in the buffers and effect of the buffers on their plasmonic properties, which define SERS capability. Performed experiments have shown that out-diffused nanoisland films are stable in PBS, in 1:100 water-diluted PBS, in the PBS-based buffer, in which NaCl is replaced by the same amount of NaClO_4_, and in acidic phosphate buffer. At the same time, exposure to PBS, in which NaCl was replaced by N(CH_3_)_4_Cl, led to the degradation of the nanoislands. In SERS experiments, substrates exposed to PBS for 22 h provided a good SERS signal from 1,2-di (4-pyridyl) ethylene (BPE) analyte, sufficient to detect a BPE monolayer.

## 2. Materials and Methods

We fabricated samples of self-assembled silver nanoisland films using the metal out-diffusion technique from a glass substrate [16]. In our experiments, we used commercial 1 mm thick soda-lime glass microscope slides “Menzel” purchased from Agar Scientific Ltd. (Stansted, Essex, UK) [17]. The glass composition is presented in Table 1.

Firstly, we introduced silver ions in the glass by silver-to-sodium ion exchange. The processing included: cleaning of the glass slides in an ultrasonic bath using acetone:isopropanol (1:1) solution and 20 min immersion of the slides in the heated to 325 °C melt containing 5 wt.% AgNO_3_ and 95 wt.% NaNO_3_ in a ceramic crucible [18]. The chemicals were purchased from Reakhim Co (Moscow, Russia). Secondly, we reduced silver ions in the glass and formed silver NPs by the annealing of the slides in hydrogen atmosphere at 250 °C for 15 min (out-diffusion process). The hydrogen was generated via electrolysis of deionized distilled water (Water deionizer Vodoley, Chimelektronika, Moscow, Russia; hydrogen generator GV-7, Meta-chrom, Yoshkar-Ola, Russia). The processing resulted in the formation of silver NPs in the bulk of the glass and nanoislands on the glass surface as reported elsewhere [16,19]. The nanoislands were observed using scanning electron microscope (SEM Supra 25, Zeiss, Oberkochen, Germany) and atomic force microscope (AFM Dimension 3100, Veeco, Plainview, NY, USA). We should note, AFM and SEM images were obtained only for qualitative visualization purpose. To verify the presence of silver in the vicinity of the surface of the prepared samples we used energy-dispersive X-ray analysis (EDX). The EDX measurements were performed using SEM JSM-7001F (JEOL, Tokyo, Japan) with EDX supplement INCA PentaFETx (Oxford Instruments, Abingdon, UK), the accuracy of the measurements being ~10%.

To characterize SPR in the NPs we measured optical absorption spectra with Specord 50 spectrophotometer (Analytik Jena GmbH, Jena, Germany). We also used optical luminescence measurements to verify the presence of molecular/ionic silver clusters in the samples, whose luminescence band lies in spectral range of 580–660 nm [20,21]. Luminescence of the samples was measured using hand-made setup that included a 365 nm LED source (LCS-0365-04-22, Mightex, Toronto, ON, Canada) and a modular spectrometer (SC82, Solar LS, Minsk, Belarus).

To test the stability of the substrates and study their interaction with several buffers, we recorded dynamic changes of the optical absorption spectra during the immersion of the samples in buffer solutions. The measurement system comprised an optical unit of Specord M400 spectrophotometer (Carl Zeiss, Oberkochen, Germany), a bath thermostat with at-cell temperature sensor and a computer controller. Firstly, a glass slide with silver nanoisland film was placed in a standard 1 cm fused silica cell, which was equipped with polytetrafluoroethylene (PTFE) adaptors so that the light beam passed through the sample almost perpendicularly to its surfaces. Then the cell was filled up by the fixed volume of a buffer (2 mL) using a plastic syringe and sealed by a paraffin film, the changes of absorption spectrum in the range 330–830 nm (transparency window of the used glass) were monitored for ~24–28 h (“the run”). Measurement interval increased from 1.2 min (collection time of 1 spectrum) to 20 min from beginning to the end of the run, about 100 spectra were collected in total. Cell temperature was maintained at 37.0 ± 0.5 °C during all the measurements. In some experiments, after the sample was in the buffer solution certain amount of time, we collected 20 µL of the solution from the cell using a plastic syringe to evaluate silver concentration in the buffer, the cell assembly was not moved during this operation. Silver concentration was measured by graphite furnace atomic absorption spectrometry (ZEEnit 650P spectrometer, Analytik Jena, Jena, Germany), limit of detection (LOD) being ~10 nM. After the dynamic optical measurements, the glass samples were cleaned by dipping them into a large volume of distilled water, and then dried and used for further characterization. Most buffers were prepared prior the measurements from adjusted Na_2_HPO_4_/KH_2_PO_4_ 0.1 M stock solution (kept at 4 °C) and the required salts, and kept at 37.0 °C for 4–6 h to equilibrate with air. Phosphate salts and tetramethylammonium chloride (N(CH_3_)_4_Cl, TMA chloride, TMAC) were purchased from Sigma-Aldrich; NaCl, NaClO_4_, and phosphoric acid was purchased from Reakhim Co. (Moscow, Russia) Aliquots of buffers were taken to measure pH of the solutions, measured results were close to the calculated values, and therefore, there was no need in the buffers adjusting. The buffers’ compositions were designed to test the relative importance of Na^+^, Cl^−^, H^+^, and OH^−^ ions in the typical PBS buffer, widely used in biochemical studies, and their influence on silver nanoislands-PBS interaction. The compositions of tested buffers are given in Table 2.

To characterize SERS applicability of the substrates, we used Raman confocal microscope (Alpha 300R, WITec, Ulm, Germany) equipped with 10×/0.25, 50×/0.8 and 100×/0.9 objectives and CW green laser (532 nm), which provided 33 mW maximal power. The objective magnification did not affect the Raman spectra and therefore we used 10×/0.25 objective to average SERS signal over ~3 µm spot, thus, eliminating the influence of the signal spikes.For SERS measurements we used a well-known analyte BPE, which is nonresonant at 532 nm [22,23]. The ~0.5 µL droplet of 10^−4^ M water solution of BPE was dried on the surface of the nanoisland films, and about 20 SERS measurements were made in the center of the dried droplet. The integration time was 1 s per measurement and the laser power was ~0.7 mW. To collect Raman spectrum (in contrast with surface enhanced Raman spectrum) of BPE, we dried the same droplet on the surface of the pristine glass (without any NPs). The drying resulted in BPE crystallization, the crystallites being ~1 µm in size. The Raman spectrum was averaged over 20 measurements across different crystallites, the integration time was 1 s, and laser power was ~33 mW. Note, the surface concentration of BPE in SERS measurements, considering uniform distribution of molecules over the area of the dried droplet on the nanoisland films, was about a monolayer, ~10^12^ mol/mm^2^ [24], while Raman spectrum was collected from at least ~3000 monolayers in ~1 µm BPE crystallite using ~50 times higher laser power.

## 3. Results and Discussion

### 3.1. Silver Nanoislands and Nanoparticles Behavior

**Figure 1:**
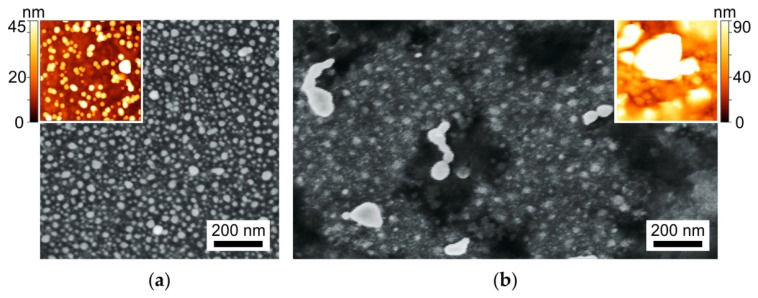
SEM and AFM images of silver nanoislands formed on the glass surface via out-diffusion before (**a**) and after 1 day exposure to PBS (**b**).

SEM and AFM images of nanoislands formed on the surface of the prepared samples are presented in Figure 1a.

**Figure 2:**
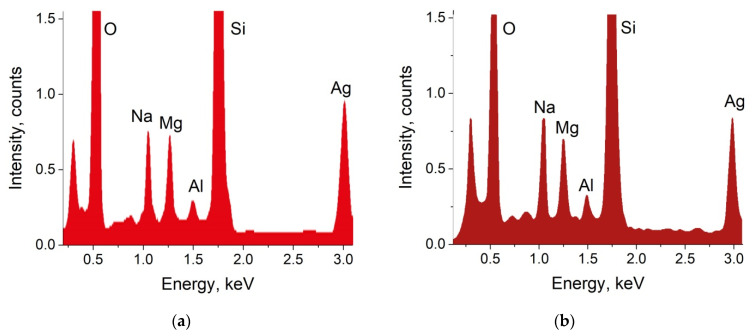
EDX spectra obtained from the prepared sample (**a**) and the same sample after 1 day exposure to PBS (**b**).

The results of the EDX analysis of the as-prepared sample is presented in Figure 2a. The peak related to silver is clearly seen in the spectrum, and calculated concentration of silver in the sample equals to 2.74 at.%.

**Figure 3:**
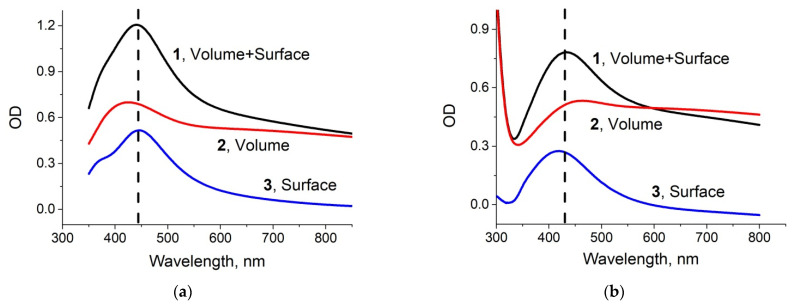
Optical absorption spectra of the prepared samples before (**a**) and after (**b**) 1 day exposure to PBS. 1—spectrum of the sample, 2—spectrum of the sample after removal of NPs from the surface (“volume” NPs spectrum), 3—residual spectrum (“surface” NPs spectrum).

The optical absorption spectrum of ion-exchanged glass slides annealed in hydrogen atmosphere (as prepared samples) is presented in Figure 3a (denoted #1). The presence of the silver NPs manifested itself with a peak at ~440 nm, this wavelength falls in the range corresponding to SPR in silver NPs [25]. We separated out spectra of the nanoparticles placed in the glass volume (spectrum #2 in Figure 3a) and nanoislands placed on the glass surface (spectrum #3) using mechanical removal of the nanoislands from the surface and subtracting the corresponding spectrum from the initial one (#1), see Figure 3a.

To test the stability of substrates in buffers used in biological studies, we kept them for 24 h in a standard PBS solution at room temperature. Slight discoloration of the exposed samples was visually observed. SEM and AFM images of the PBS-exposed sample are presented in Figure 1b. It can be seen that the scale of the surface relief has increased at least twice, and the nanoislands are covered with “flakes” of another substance. To check for the presence of nanoislands after exposure to PBS, we performed EDX characterization, re-measured the optical absorption spectrum, and, as before, separated the contributions of volume and surface NPs in optical absorption of the sample. Obtained EDX spectrum is presented in Figure 2b, and the optical absorption spectra are shown in Figure 3b. The results of the measurements were quite unexpected: silver still presented in the vicinity of the glass surface, and after PBS exposure its content is 2.34 at.% (compare with 2.74 at.% before the exposure), and the optical absorption peak corresponding to the surface nanoislands remained unchanged, however the volume NPs peak essentially decreased. It means that the surface NPs are resistant to PBS solution, while NPs in the bulk of the sample dissolve. A small difference in silver concentration measured with EDX can be related with the penetration of e-beam in the subsurface region of the glass initially containing silver NPs, which dissolved after exposure to PBS.

**Figure 4:**
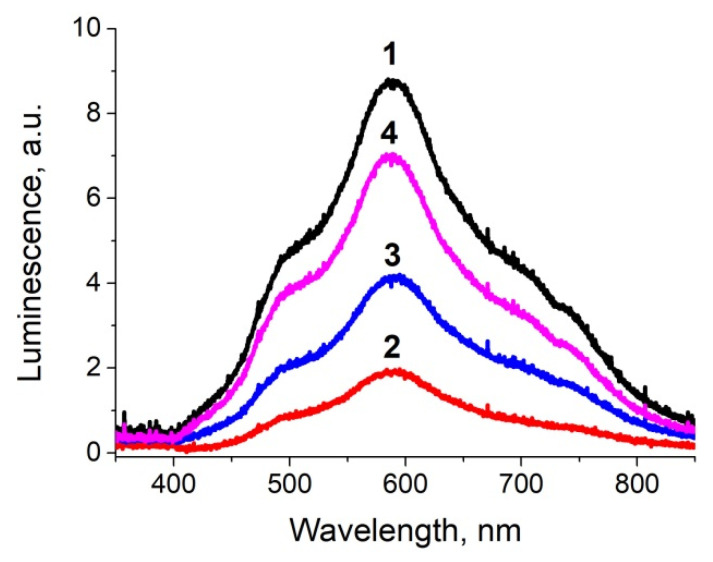
Luminescence spectra of the glass: 1—after silver to sodium ion exchange, 2—after the ion exchange and annealing in hydrogen (glass with Ag NPs), 3—after 1-day exposure to PBS, 4—after 4-day exposure to PBS. The excitation wavelength was 365 nm.

The luminescence spectra of the ion-exchanged glass, glass after the ion exchange and annealing in hydrogen, and the samples with silver NPs after exposure in PBS for 1 and 4 days are presented in Figure 4. All spectra demonstrate a luminescence peak at about 590 nm corresponding to the presence of silver ionic clusters-(Ag_3_)^2+^ trimers [26,27,28], whose concentration is in equilibrium with the concentration of silver ions in glasses and increases with the concentration of the latter. The glass subjected to ion exchange contains the largest amount of silver ions; thus, the luminescence has maximal amplitude. Processing in hydrogen leads to the reduction of silver ions to a neutral state and coalescence of the clusters to silver NPs; this manifests itself in a drop of silver ionic clusters luminescence peak. However, after exposure of the NPs-containing glass to PBS solution, the peak begins to increase, and its amplitude grows with the duration of PBS exposure. This reflects the reappearance of silver ions and, respectively, (Ag_3_)^2+^ clusters in the samples. It can only be associated with the dissolution of NPs in the bulk or on the surface, for there are no other sources of silver ions. Similar increase in cluster luminescence corresponding to a decrease in the magnitude of SPR related to NPs in the bulk of a glass was recently observed in our experiments on a laser irradiation of a silver-containing glass [29]. Since the optical absorption peak corresponding to surface-placed NPs does not change (see Figure 3b), the dissolution of silver NPs occurs in the bulk of the sample. As far as we know, this phenomenon was observed for the first time.

### 3.2. Influence of Buffer Composition

**Figure 5:**
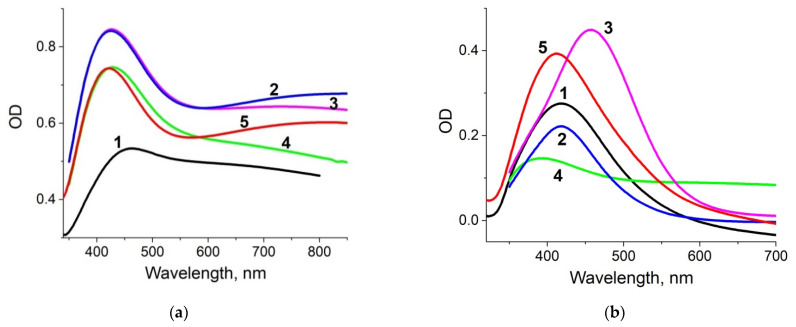
Optical absorption spectra components related to “volume” (**a**) and “surface” (**b**) NPs in the prepared samples exposed to different buffers (marked with the buffers #, see Table 2) for 24 h.

The dissolution of NPs in the subsurface region of the sample, (“volume” NPs), revealed in our experiment can be associated with the interaction of ions contained in PBS solution with silver in the bulk, silver on the surface, or with the formation of chemical compounds. We did the similar experiments with other buffers, which are listed in Table 2. The results illustrating spectral properties of our substrates after interaction with different buffers are shown in Figure 5.

The changes in the “volume” NPs SPR absorption peak are registered only for PBS buffer, this allows us to put a hypothesis about the role of chloride ions in the dissolution of NPs in the bulk. At the same time, Cl^−^ containing TMAC buffer, unlike PBS, dissolves nanoislands on the surface (see spectrum #4 in Figure 5b). As for other buffers, NPs on the surface “survive” in the solutions. All the spectra measured after removal of NPs from the glass surface (“volume” spectra) demonstrate a big “shoulder” at longer (red) wavelength range after exposure to the buffer. Most pronounced this “shoulder” is for diluted PBS (buffer #2) and acidic buffer (buffer #5). We suppose this is due to the appearance of atomic silver in the glass.

**Figure 6:**
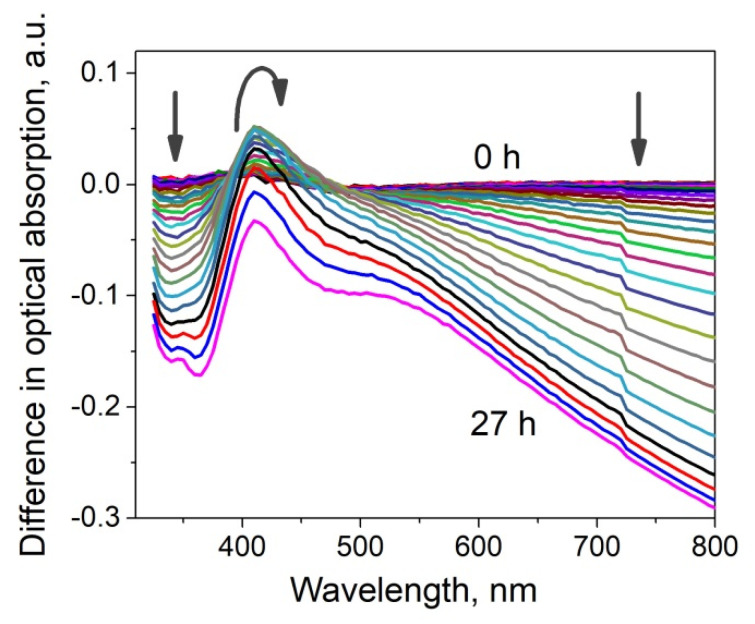
Dynamic changes of differential absorption spectrum of NPs-containing glass immersed in 2 mL of PBS buffer at 37 °C. The difference from the first spectrum of the kinetic series (45 s from the beginning of the reaction) is plotted. The spectra are taken with 1-h interval (0–27 h), arrows indicate the direction of changes.

Dynamic changes of absorption spectrum of the glass samples were observed in all the experiments, but the magnitude of changes depended strongly on the composition of the buffer. Figure 6 gives a representative example of the changes of absorption spectrum of the glass containing Ag NPs- in PBS buffer during a 27-h immersion. The spectral variance comprised changes of magnitude and shape of SPR peak; the profound decrease of absorption in the red region was observed only in PBS buffer. Exponents or other simple formal kinetic models poorly described the shape of kinetic curves, so no rate analysis was attempted. Table 3 presents qualitative description of the behavior of the samples in different buffers.

**Figure 7:**
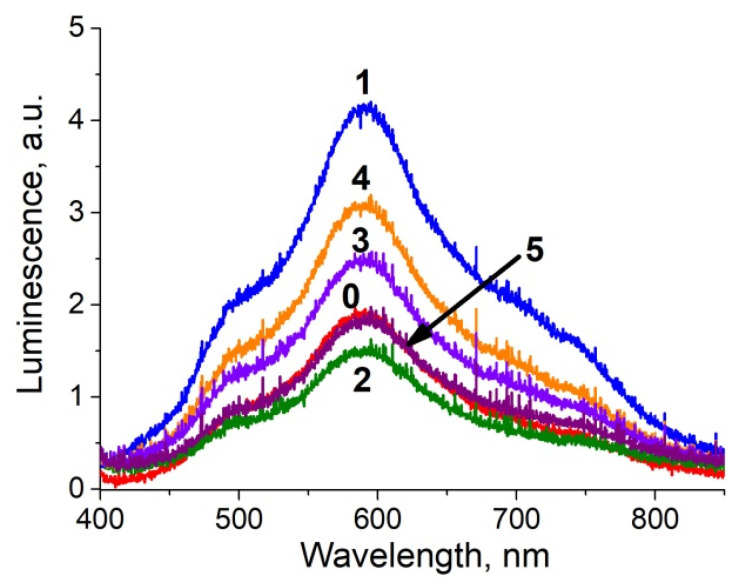
The luminescence spectra of Ag NPs-containing glass after exposure to different buffers. The excitation wavelength was 365 nm, the curves are marked according to the buffers ##, #0 corresponds to the untreated sample.

The luminescence spectra characterizing the presence of silver clusters (supposedly (Ag_3_)^2+^ [26]) in the substrates are shown in Figure 7 (also see Table 3). The acidic buffer almost does not change the concentration of silver in the substrates in relation to the glass containing NPs (annealed ion-exchanged glass). When using a diluted PBS, changes in concentration are minimal; perhaps, some of the ions reduce, and this increases absorption in longer (red) wavelength range (see Figure 5a, spectrum #2). The other buffers increase the concentration of silver ions in the samples, but for different reasons. In the case of PBS, silver NPs dissolve in the volume (see Figure 3), immersion in TMAC buffer leads to dissolution of NPs on the surface (see Figure 5), both result in an increase of the concentration of silver ions.

### 3.3. Chemical Conversions

The detailed mechanism of the chemical conversion of silver in the film needs further studies; however, some ideas may be proposed basing on qualitative behavior of the samples after exposure to various buffers. The driving force for the chemical conversion is the shift of Ag_s_↔Ag(I) + e^−^ equilibrium by the stabilization of Ag(I) state by anions in the solution and/or in Ag(I) salts. The electrons remain in metallic NPs, decreasing their charge and redox potential, and can further pass to the available oxidizers, most probably, dissolved dioxygen. Silver ends up in the “insoluble” salts, so the process is not limited by buffer saturation. Silver concentration in the buffer is determined by formation of soluble Ag(I) species. The measured values in the PBS and PB⋅TMAC buffers after 24 h of the reaction ([Ag] 3–5 μM) correspond in the order of magnitude to AgCl/[Cl^−^] equilibrium values predicted by PREEQC aqueous model [30]: for [Cl^−^] 0.15 M; total saturation concentration for [Ag(I)] species is 5.6 μM at 20 °C and 18 μM at 37 °C, which corresponds predominantly to [AgCl_x_]^x−1^ complexes. Ignoring temperature and ionic strength effects, equilibrium redox potentials, *E*, can be roughly estimated by Nernst equation. As expected, chloride ions have the largest affinity to Ag(I): *E* 0.27 V for 0.15 M Cl^−^ (PBS and PB⋅TMAC buffers), *E* ~ +0.46 V for 0.15 mM Cl^−^ (diluted PBS). Phosphate has much smaller affinity (*E* ~ +0.53 V for 0.012 M [PO_4_]^3−^), so does carbonate from dissolved CO_2_; in the presence of chloride, their influence on silver reactions may be ignored. Aqueous species, perchlorates, and oxides of Ag(I) are even less stable having *E* ~ +0.8 ÷ +2.0 V at pH 7.0 [31] and may be excluded from consideration in all cases studied. It is, thus, not surprising that chloride-containing buffers affect the film most actively. This results in stronger dissolution of metallic silver and appearance of more [Ag_3_]^2+^ species in the glass (see Figure 7). Probably, in buffers with low chloride, the electrochemical potential of NPs is not sufficiently low for notable oxidation. Ag_s_/AgCl reaction is known to have small activation potential, so the processes are likely to be limited by oxidation and diffusion. In fact, about 0.2 μM of soluble silver could be detected even in diluted PBS buffer 30 s after its addition to the glass sample. Surprisingly, the pattern of NPs degradation (bulk/surface) in the buffers with the same chloride concentration depended on cation composition. In high sodium PBS buffer mostly the bulk Ag species were oxidized, while low sodium PB⋅TMAC buffer affected only the surface NPs. This effect has no obvious explanation; however, it should be noted that sodium ions can enter the near-surface volume of the glass, while TMA ions cannot. There are several hypothetic ways in which sodium ions may affect NPs oxidation. First, they may compete with positively charged Ag species and alter their chemical potential. The gross displacement of silver species from the glass by more energetically favorable sodium ions may be the driving force of the process. Second, sodium ions may facilitate migration of silver from bulk NPs to surface NPs, e.g., in the form of [Ag_3_]^2+^, making it accessible for oxidation.

### 3.4. Surface Enhanced Raman Scattering

**Figure 8:**
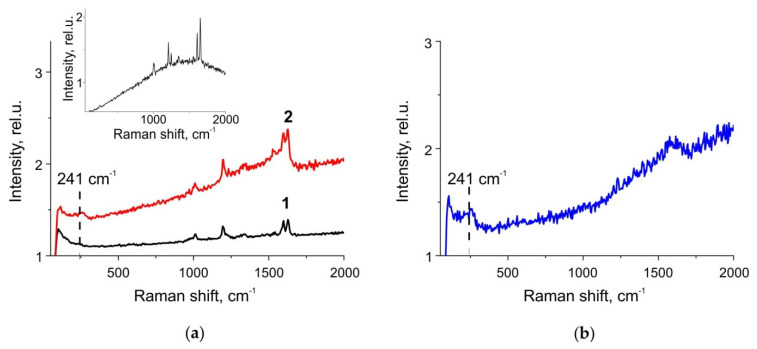
SERS spectra of a monolayer of BPE deposited on a silver nanoisland film before (1) and after (2) exposure to PBS (**a**). The inset: Raman spectrum from BPE crystallites on glass without NPs. Raman spectra of a pristine silver nanoisland film (without BPE) after exposure to PBS (**b**).

Finally, we have verified the applicability of the substrates for SERS measurements. Since the enhancement of the Raman signal is provided by NPs on the surface, not in the bulk, it was natural to expect a stable to PBS SERS signal from the samples under study. SERS measurements of BPE deposited on substrates with out-diffused silver nanoislands exposed to PBS solution for 22 h showed a stable Raman signal corresponding to BPE and a significant signal enhancement compared to unprocessed glass (see Figure 8a). BPE characteristic lines at 1010, 1194, 1333, 1538, 1598, 1631 cm^−1^ are repeated in the BPE spectra from the unprocessed silver nanoisland film and from the film after immersion to PBS. It is worth noting that the background of the spectrum measured after exposing the nanoislands to PBS (line 2 in Figure 8a) is higher than the background of the spectrum obtained from slide with nanoislands that were not exposed to PBS (line 1). Moreover, the same background one can see in the spectrum of a pure (no analyte) silver nanoisland film exposed to PBS (Figure 8b). This is because of the increase in the luminescence of silver clusters [20,21] after the exposure, which is illustrated by Figure 4 and explained by the formation of the clusters when nanoparticles grown in the bulk of the glass dissolve. Besides, in the SERS spectrum of the BPE deposited onto the glass with silver nanoisland film after the PBS exposure, a line appears at 241 cm^−1^. The same line also presents in Raman spectrum of the pure silver nanoisland film exposed to PBS. We associate this line with the formation of AgCl [32], which can be seen also in our SEM and AFM measurements as “flakes” on top of the NPs (see Figure 1b). The increase in the range 1250–1750 cm^−1^ visible both in the spectra of PBS-treated silver nanoisland film itself (Figure 8b) and in the BPE spectra collected using the PBS-treated nanoisland film (Figure 8a) may correspond to amorphous carbon [33] originated from, e.g., reduction of organic impurities.

Using the measured spectra, we calculated the enhancement factor for nanoisland silver films under study in accordance with the procedure described in Ref. [13]. The enhancement factor was ~4 × 10^4^ for the nanoisland film before PBS, and ~5 × 10^4^ for the film after PBS exposure. Thus, exposure in PBS does not reduce the SERS signal, and the films can be effectively used for Raman measurements using PBS buffer.

## 4. Conclusions

It was shown that silver nanoisland films obtained by out-diffusion of silver from glass exhibit good stability in many buffers, including the most common in biological research PBS, 1:100 water-diluted PBS, PBS with the replacement of NaCl by NaClO_4_, and acidic phosphate buffer. At the same time, the replacement of NaCl by N(CH_3_)_4_Cl leads to the degradation of silver nanoislands, which can be attributed to the effect of chlorine ions. After exposure to PBS, the nanoisland films used as substrates for SERS provide a good signal from a monolayer of BPE, the surface enhancement factor being ~5⋅10^4^. This makes the out-diffused silver SERS substrates prospective for biological applications, including real-time measurements and biosensors. Additionally, we registered for the first time the dissolution of silver NPs in the bulk of the samples exposed to PBS, while NPs on the glass surface remained unchanged. We associate this phenomenon with the interaction of ions contained in PBS solution with silver, which results in the shift of corresponding chemical equilibrium. The dissolution of silver NPs in the bulk of the samples immersed in PBS does not influence the sensor applicability of the studied substrates.

## Figures and Tables

**Table 1:** Composition of “Menzel” glass in wt.% of oxides.

SiO_2_	Al_2_O_3_	Na_2_O	K_2_O	MgO	CaO	Others
72.2	1.2	14.3	1.2	4.3	6.4	0.33

**Table 2:** Chemical composition and active ions of tested buffers.

Buffer Number (#)	Name	Composition ^1^	Active Ions
1	PBS	NaCl 137 mM, Na_2_HPO_4_/KH_2_PO_4_, [PO_4_] = 12 mM, pH 7.4	Na^+^, Cl^−^
2	water (diluted PBS)	NaCl 0.137 mM, Na_2_HPO_4_/KH_2_PO_4_, [PO_4_] = 0.012 mM, pH 7.1	H^+^, OH^−^
3	PB⋅NaClO_4_	NaClO_4_ 137 mM, Na_2_HPO_4_/KH_2_PO_4_, [PO_4_] = 12 mM, pH 7.4	Na^+^
4	PB⋅TMAC	N(CH_3_)_4_Cl 137 mM, Na_2_HPO_4_/KH_2_PO_4_, [PO_4_] = 12 mM, pH 7.4	Cl^−^
5	acidic phosphate buffer	H_3_PO_4_/Na_2_HPO_4_, [PO_4_] = 12 mM, pH 3.3	H^+^

^1^ Measured pH values (measurement uncertainty 0.1–0.2 pH units) are specified.

**Table 3:** Qualitative behavior of the samples after exposure to the buffers for 24 h.

Change after Exposure to the Buffer	Buffer				
	PBS	Diluted PBS	PB⋅NaClO_4_	PB⋅TMAC	Acidic Buffer
Degradation of “surface” NPs	−	−	−	+	−
optical absorption input ^1^					
Degradation of “volume” NPs	+	−	−	−	−
optical absorption input					
Visible discoloration	+	−	−	+	−
and tarnishing of the film					
Overall spectral changes in 330–830 nm range, peak, |ΔD_24h_| ^2^	Strong 0.35	Weak	Weak	Strong	Strong
		0.04	0.02	0.22	0.2
Change in NPs SPR peak, |ΔD_24h_|_max_	Decrease	Shape change	Shape change	Decrease	Decrease
	0.17	0.04	0.04	0.22	0.19
Change in red (600–900 nm) absorption	Strong decrease	Very small decrease	Very small	Small	Decrease
shoulder ^3^			decrease	decrease	
Change in luminescence at 590 nm	Strong increase	Small	Small	Strong	Very small
	2231	decrease	increase	increase	decrease
		−400	563	1172	−75
Silver concentration in buffer	3.26	2.8	0.86	4.99	not
after 22–24 h of the exposure, μM					measured

^1^ obtained using subtracting of the spectrum measured after mechanical removal of the NPs from the initial spectrum; ^2^ ΔD_24h_—change of film/glass absorption during ~24 h of the dynamic study; ^3^ since inputs of “surface” and “volume” NPs can hardly be distinguished in the course of the kinetic spectral measurements, only phenomenological description is presented.
